# The interplay between gingival crevicular fluid microbiome and metabolomic profile in intensively treated people with type 1 diabetes - a combined metagenomic/metabolomic approach cross-sectional study

**DOI:** 10.3389/fendo.2023.1332406

**Published:** 2024-02-02

**Authors:** Iwona Gregorczyk-Maga, Michał Kania, Michalina Dąbrowska, Emilia Samborowska, Natalia Żeber-Lubecka, Maria Kulecka, Tomasz Klupa

**Affiliations:** ^1^ Institute of Dentistry, Faculty of Medicine, Jagiellonian University Medical College, Krakow, Poland; ^2^ Chair of Metabolic Diseases and Diabetology, Faculty of Medicine, Jagiellonian University Medical College, Krakow, Poland; ^3^ Doctoral School of Medicine and Health Sciences, Jagiellonian University Medical College, Krakow, Poland; ^4^ Department of Genetics, Maria Sklodowska-Curie National Research Institute of Oncology, Warsaw, Poland; ^5^ Mass Spectrometry Laboratory, Institute of Biochemistry and Biophysics, Polish Academy of Sciences, Warsaw, Poland; ^6^ Department of Gastroenterology, Hepatology and Clinical Oncology, Center of Postgraduate Medical Education, Warsaw, Poland; ^7^ Center of Advanced Technologies in Diabetes, Chair of Metabolic Diseases, Faculty of Medicine, Jagiellonian University Medical College, Krakow, Poland

**Keywords:** type 1 diabetes, continuous subcutaneous insulin infusion, gingival crevicular fluid, microbiome, metabolome, gingivitis

## Abstract

**Aims:**

This study aimed to assess the gingival crevicular fluid (GCF) microbiome and metabolome of adults with type 1 diabetes (T1D) treated with continuous subcutaneous insulin infusion (CSII).

**Methods:**

In this cross-sectional study, the GCF of adults with T1D treated with CSII and non-diabetic controls were sampled, and metagenomic/metabolomic analyses were performed.

**Results:**

In total, 65 participants with T1D and 45 healthy controls with a mean age of 27.05 ± 5.95 years were investigated. There were 22 cases of mild gingivitis (G) in the T1D group. There were no differences considering the Shannon and Chao indices and β-diversity between people with T1D and G, with T1D without G, and healthy controls. Differential taxa were identified, which were mainly enriched in people with T1D and G. Acetic acid concentration was higher in people with T1D, regardless of the presence of G, than in healthy controls. Propionic acid was higher in people with T1D and G than in healthy controls. Isobutyric and isovaleric acid levels were higher in individuals with T1D and G than in the other two subgroups. The concentration of valeric acid was lower and that of caproic acid was higher in people with T1D (regardless of gingival status) than in healthy controls.

**Conclusions:**

The identification of early changes in periodontal tissues by targeting the microbiome and metabolome could potentially enable effective prevention and initial treatment of periodontal disease in people with T1D.

## Highlights

- To our knowledge, this is the first study to investigate the GCF microbiome in people with T1D. The study population was treated with modern technologies, such as CSII, demonstrating good glycemic control.
- Intensively treated people with T1D with satisfactory glycemic control and non-diabetic individuals generally showed good oral and periodontal health.
- With relatively well-controlled diabetes, slight differences in glycemic control did not significantly affect the oral microbiome, which was comparable to that observed in people without diabetes.
- Despite good metabolic control of diabetes, people with T1D in our study had a higher prevalence of mild gingivitis than healthy controls. This subpopulation exhibited shifts in the GCF microbiome and metabolome, resembling those in periodontitis.

## 1 Introduction

The oral cavity, also known as the mouth or buccal cavity, is the first section of the digestive system (1–3). It consists of several distinct microbial niches, such as tooth surfaces, gingiva, gingival sulci, and mucosal surfaces of the tongue, cheeks, lips, and palate (4). Species residing in the oral cavity are regarded as part of the oral microbiome (5), which is one of the most important microbial complexes in humans (6). It has been reported to include over 1000 species of bacteria, with a few lesser-known taxa emerging from the most recent studies, archaea, which are less abundant and diverse than bacteria, approximately 100 species of fungi, and a rich virome (7). Although the oral microbiome is defined as all microorganisms residing in the human oral cavity and its extensions (reaching the distal part of the esophagus), most studies have focused on samples obtained from the oral cavity itself (4). They can have both positive and detrimental effects on general health and the local state of the oral cavity (5, 6). The link between the oral microbiome and organisms is bidirectional. Diseases affect microbial composition and function, and microorganisms modify their susceptibility to disease states, course, and prognosis. One such condition is diabetes (8–10).

Type 1 diabetes (T1D) is a chronic autoimmune disease in which pancreatic beta cells responsible for insulin production are destroyed. People with T1D account for 5–10% of the population with diabetes (11). Continuous subcutaneous insulin infusion (CSII) using an insulin pump is one of the most notable advancements in diabetes technology. Insulin pump therapy has become the preferred treatment for T1D, as it mimics the physiological secretion of insulin better than multiple daily injections. People with T1D treated with CSII therapy compared to traditional multiple daily insulin injections achieve improved glycemic control (12).

Diabetes not only affects the oral microbiome but also increases the risk of multiple local oral abnormalities in the oral cavity, affecting the quality of life of people with diabetes (13). People with diabetes are highly susceptible to dental caries, tooth loss, and periodontal disease (PD) (14, 15). The mechanisms responsible include quantitative and qualitative salivary changes, formation of advanced glycosylation end products, and their deposition in tissues, leading to vascular dysfunction due to hyperglycemia and accompanying atherosclerosis (13, 16).

Alterations in the oral microbiome of people with diabetes have been extensively investigated; however, most studies have focused on type 2 diabetes. The oral microbiome of individuals with T1D has rarely been the subject of extensive research. Interestingly, the results often contrast and cannot be generalized. Microbiome shifts have been reported to affect the immune function and metabolic control in this population (17, 18). Moreover, reports tend to focus on those with poor oral health, caries, or PD (19, 20).

Subgingival plaque accumulation is associated with the supragingival environment (21). Caries and PDs, which are common oral biofilm-related diseases, are caused by resident microorganisms in the oral cavity (22). The *red complex* is a specific group of bacteria considered to play a major role in the development of adult PD. These bacteria include *Porphyromonas gingivalis, Treponema denticola*, and *Tannerella forsythia* (23). People with T1D and good metabolic control of diabetes without a history of oral pathologies, are still underreached, showing alterations in the oral microbiome, such as a greater abundance of *Streptococcus* spp., *Actinomyces* spp., and *Rothia* spp., than healthy controls (17). Additionally, some studies have implemented traditional bacterial identification methods (24, 25). Therefore, the oral microbiome signature in T1D has not yet been established.

Novel approaches to the holistic investigation of phenomena include metagenomics, transcriptomics, proteomics, and metabolomics to identify the differences between healthy and diseased participants and their underlying mechanisms (26–28). Metabolomics can target various environments, such as stools, saliva, and gingival crevicular fluid (GCF), metabolites and metabolomics which can be associated with several diseases, including diabetes and PD (29–32). The roles of various metabolites in the human body, such as in the gut, respiratory tract, genitourinary tract, and oral cavity, are complicated and not well understood. In some cases, different concentrations of the same metabolite can have opposite effects depending on its location. Moreover, metabolites delivered in nutrients can influence microbiome composition, but these substances are also produced by bacteria, implicating complex bidirectional relationships (33).

Usually, saliva has been investigated, with only a few studies assessing the GCF microbiome or its metabolome. The GCF, which is derived from periodontal tissues, plays an important role in preserving the junctional epithelium and other periodontal structures (34, 35). It may play a dual role – either maintaining periodontal health and assuring the antimicrobial defense of the periodontium, as it contains immune cells, antibodies, and cytokines, or, when altered by acute or chronic immune processes, is responsible for the emergence of PD (36, 37). GCF contains multiple proteolytic and hydrolytic enzymes, bone-related biomarkers, cell death, and tissue breakdown products. Oral bacteria and products of their metabolism can also be identified in the GCF and add to the complexity of this oral niche (35). Thus, GCF analysis has the potential to become a predictive, preventive, and personalized medical approach for the diagnosis of PDs. Although GCF is inherently associated with PD, its metabolomics have rarely been investigated (29).

The present study aimed to assess the oral GCF microbiome and metabolome status in the group of adult people with T1D, homogenous with respect to the mode of diabetes management (CSII) and glycemic control.

## 2 Methods

### 2.1 Study design and participants

This was a cross-sectional study consecutively that recruited 110 adult participants. Sixty-five people with T1D were treated with continuous subcutaneous insulin infusion (CSII) in the Outpatient Clinic of the Department of Metabolic Diseases and Diabetology of the University Hospital in Krakow, an academic referral center for diabetes in southeastern Poland. Patients were matched with 45 non-diabetic controls.

Between October 1 and December 31, 2022, patients attending the clinic who met the inclusion criteria were offered the opportunity to participate in the study. After obtaining written consent, the sampling date was set and participants were instructed on how to prepare for the study procedures. The inclusion criteria were age 18–35 years, T1D diagnosed at least 1 year before recruitment, treatment with CSII for at least 6 months, and informed consent to participate. The exclusion criteria were pregnancy or breastfeeding and comorbidities, such as metabolic syndrome, cardiovascular disease, cancer, severe liver failure, or kidney failure. Diagnosis of T1D was confirmed based on the Diabetes Poland criteria. Data on age, sex, comorbidities, diabetes duration (on the day of sampling), glycated hemoglobin (HbA1c), and T1D treatment were extracted from the medical records. HbA1c levels were measured using high-performance liquid chromatography. Individuals with diabetes were matched with non-diabetic controls. The ratio of controls per case was set at 0.7:1.

Information on daily oral hygiene routines, frequency of dental appointments, and history of dental procedures were recorded. Examination of the oral cavity was performed by a trained dentist in a specially prepared room equipped with a dental chair and shadowless lamp to ensure maximal privacy for the participants. During periodontal examination, a WHO 621 periodontal probe was used to assess the Gingival Index (38), Gingival Sulcus Bleeding Index (39), and Plaque Index (39). PerioCP probe-15 was utilized to assess the Clinical Attachment Level (CAL) and Pocket Probing Depth. The oral health status was assessed using the Oral Hygiene Index (40), Community Periodontal Index, and Treatment Needs (41).

Microbiological samples were collected from the oral cavity by refraining from brushing the teeth with any kind of toothpaste or rinsing the oral cavity with any kind of mouthwash for 12 h prior to the visit. Additionally, information on the previous use of selected types of oral health products (i.e., toothpaste containing triclosan, mouthwash containing chlorhexidine, or any oral topical agent) was recorded. On the day of the examination, the participants refrained from brushing their teeth and drinking, eating, or smoking for 1 h before the microbiological samples were collected. To prevent salivary contamination of GCF, pieces of sterile gauze were used to remove excess saliva from the mucosal and dental surfaces. PerioPaper Strips were used to collect GCF samples. The strips were placed in the gingival pocket for 30–45 s until the surface was soaked. After the collection, strips were placed in 1 mL of Liquid Amies in a plastic screw cap tube (COPAN ESwab™).

### 2.2 DNA extraction and 16S rRNA sequencing

Genomic DNA was extracted and purified from PerioPaper Strips using the QIAamp DNA Mini Kit (QIAGEN, Hilden, Germany) with modifications to the bacterial protocols. DNA purity was measured on a NanoDrop™ 2000 Spectrophotometer (Thermo Fisher Scientific, Waltham, MA, USA) and quantified using fluorimetry with the Qubit dsDNA High Sensitivity Assay (Thermo Fisher Scientific, Carlsbad, CA, USA). Bacterial 16S rRNA libraries were prepared using an Ion 16S™ Metagenomics Kit and an Ion Plus Fragment Library Kit as previously described (42). Next, constructed libraries were sequenced on an Ion Torrent Personal Genome Machine (PGM) platform (Thermo Fisher Scientific, Waltham, MA, USA) using Ion PGM™ Hi-Q™ View Kit (Thermo Fisher Scientific, Waltham, MA, USA).

### 2.3 GCF microbiome metabolite quantification

The concentrations of short-chain fatty acids (SCFAs) and trimethylamine derivatives were determined using liquid chromatography coupled with mass spectrometry (Waters Acquity Ultra Performance Liquid Chromatograph, Waters TQ-S triple-quadrupole mass spectrometer, Waters). Waters MassLynx software was used for instrument control and data acquisition. Waters TargetLynx was used to process the data. To evaluate metabolites’ concentrations, one strip with gingival crevicular fluid (PerioPaper Strip) was incubated with 50 µL PBS for 30 min to extract all analytes. SCFAs and lactic acid analysis were based on derivatization using 3-nitrophenylhydrazine and N-(3-dimethylaminopropyl)-N^′^-ethylcarbodiimide-pyridine solution. LC-MS/MS analysis was performed in the negative electrospray ionization multiple-reaction monitoring mode. The SCFAs were separated using a Waters BEH C18 column (1.7 µm, 2.1 mm x 50 mm) and a Waters BEH C18 guard column (1.7 µm, 2.1 mm x 5 mm). A 1 mL of formic acid in 1 L of water was used as mobile phase A, and 1 mL of formic acid in acetonitrile was used as mobile phase B. The flow rate of the mobile phase was set at 0.6 mL/min. To determine trimethylamine (TMA), choline, carnitine, betaine and glycerophosphorylcholine, only 20 µL of a sample was used. TMA was performed using a butyl bromoacetate solution as the derivative reagent. The LC-MS/MS method has been previously described (43).

### 2.3 Statistical analysis

The null hypothesis used in the study was that there are significant differences in the alpha and beta diversity of the oral GCF microbiome between people with T1D treated with CSII and healthy non-diabetic controls.

Metagenomic analyses included comparisons between healthy controls and people with T1D and gingivitis (G) vs. those with T1D without G. Individuals with T1D were additionally divided into quartiles based on HbA1c and the first and fourth quartiles were compared. Metabolomic analyses included a comparison between healthy controls and people with T1D and G vs. those T1D without G. Additionally, we analyzed the correlation between metabolite concentrations and HbA1c%. In the metagenomic-metabolomic analysis, correlations between the identified bacterial taxa and selected metabolite concentrations were compared.

The PS Imago Pro ver. 8.0 and Statistical ver. (13) were used for all the statistical analyses. When data were missing, a complete case selection approach was used. Normality of the continuous variable distribution was assessed using the Shapiro–Wilk test. Differences between groups were analyzed using Student’s t-test or nonparametric tests (Mann–Whitney U test, Kruskal–Wallis ANOVA), when appropriate. Continuous variables were presented as arithmetic means ( $\bar{x}$ ) ± standard deviations (SD) or as the median with interquartile range (IQR) when the data were not normally distributed. The distribution of categorical variables was described as counts and percentages. Statistical testing was performed to compare categorical variables using an independent sample chi-square test or Fisher’s exact test, when appropriate. Statistical significance was set at p <0.05. The Bonferroni method was used to correct multiple comparisons. Power calculations using the RNASeqPower package estimated a power of 90% for a coverage depth of 10x, sample size of 45 (each group), coefficient of variation of 0.5, and effect size (fold-change) of 1.5.

The unmapped BAM files were converted into FASTQ files using Picard SamToFastq (44). Additional steps of the analysis were performed using the Mothur version 1.47 software (45). FASTQ files were converted to FASTA format. For the analyses, only sequences 200–300 bp in length with an average base quality of 20 in a sliding window of 50 bases and a maximum homopolymer length of 10 were used. Chimeric sequences were identified using the VSEARCH chimera detection algorithm with default parameters (46)and the internal sequence collection as the reference database. Chimeric sequences were removed and the remaining 16S rRNA sequences were classified using the Wang method and the SILVA bacterial 16S rRNA database (47) for reference (release 138) with an 80% bootstrap cut-off.

Differential taxon abundances were assessed using a mixed-effects model implemented in LinDA (48). The nonparametric Shannon diversity index and Chao1 richness index were determined using Mothur, with differences in the values of the indices assessed using the Mann–Whitney U-test. Bray–Curtis indices and principal coordinate analysis (PCoA) were performed using the vegan package (49). FDR-adjusted (50) P-values ≤ 0.05 were considered statistically significant. For SCFAs and amino acids, correlations with bacteria were determined using the Spearman’s coefficient.

### 2.4 Ethics and reporting guidelines

This study involving humans was approved by the Jagiellonian University Bioethics Committee (Komisja Bioetyczna Uniwersytetu Jagiellońskiego). The study was conducted in accordance with local legislation and institutional requirements. All the participants provided written informed consent to participate in this study.

This study was conducted in accordance with the Strengthening the Reporting of Observational Studies in Epidemiology guidelines. This checklist has been added to the **Supplementary File**.

## 3 Results

A total of 110 participants were included in the study: 65 people with type 1 diabetes and 45 matched healthy controls. The mean age of the sampled population was 27.05 ± 5.95 years. Sixty percent of participants were male. The mean duration of diabetes was 15.5 ± 8.4 years. All people with T1D were treated with continuous subcutaneous insulin infusion. The mean Hba1c% was 6.97 ± 0.95% (53 ± 2.2 mmol/mmol). No baseline differences were observed between the groups (**Supplementary Table 1**).

None of the participants had dental implants or prostheses. There were no cases of periodontitis in the study population, and 22 cases of mild G were observed in the T1D group. The selected dental indices and the prevalence of edentulism are summarized in **Supplementary Table 1**.

### 3.1 GCF microbiome analysis

**Figure 1:**
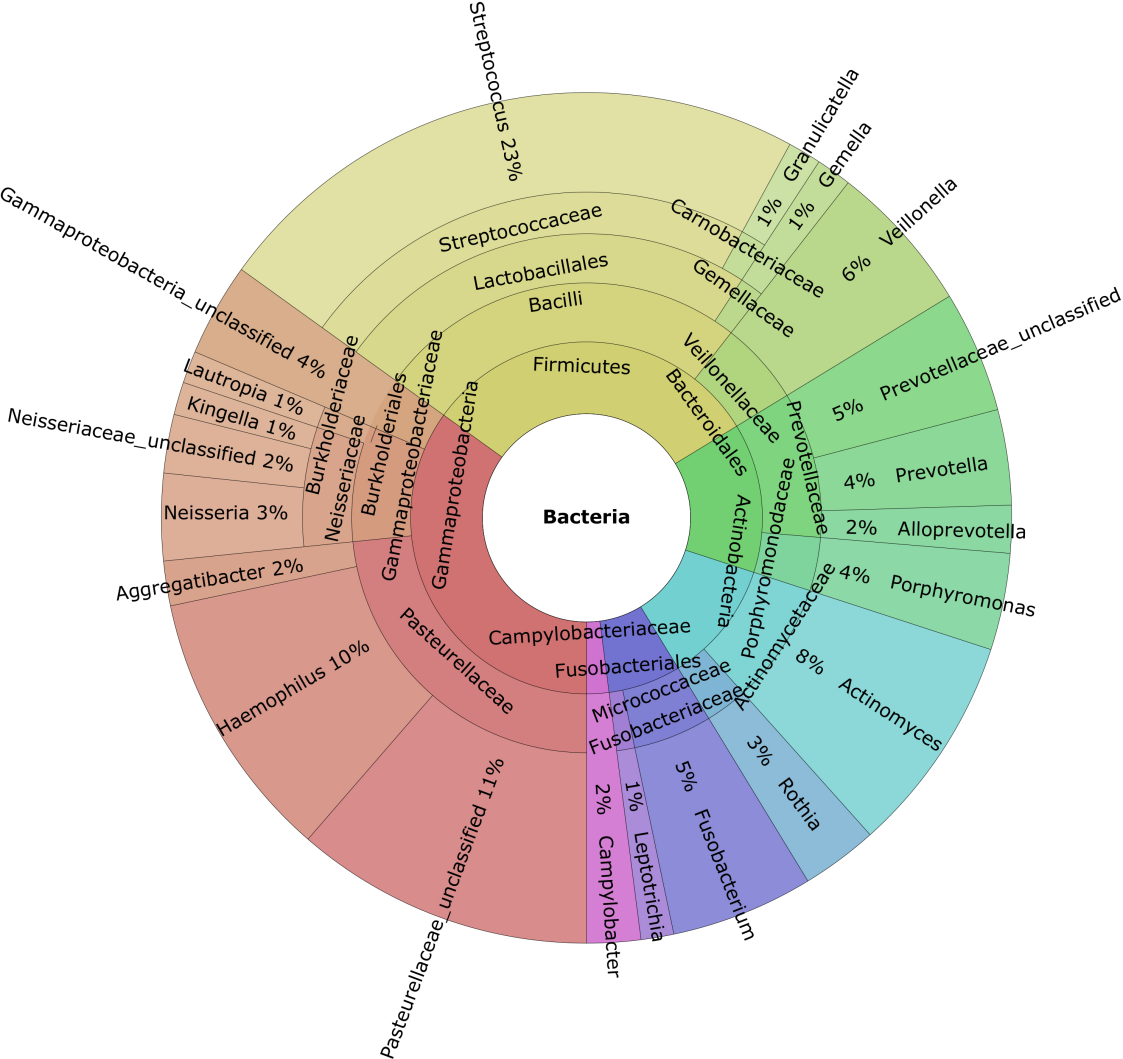
Krona charts of the genera with a mean abundance greater than 1% of the total found in the gingival crevicular fluid samples.

Taxonomic profiling revealed that *Firmicutes* was the most abundant phylum (mean: 34%), followed by *Proteobacteria* (mean: 27%), *Bacteroidota* (mean: 16%), *Actinobacteriota* (mean: 12%), and *Fusobacteriota* (mean: 7%) (**Figure 1**). At the genus level *Pasteurellaceae*_unclassified, *Haemophilus*, *Actinomyces*, *Veillonella*, and *Fusobacterium* were the most abundant genera (**Figure 1**).

To identify the potential differences in the structure of the GCF microbiome, we first evaluated the α- and β-diversity in subgroups of T1D cases and controls divided according to clinical status. The α-diversity was analyzed using the Shannon index, a marker of bacterial richness and evenness, and the Chao index, a marker of richness. The β-diversity was analyzed using PCoA of Bray-Curtis distances.

**Figure 2:**
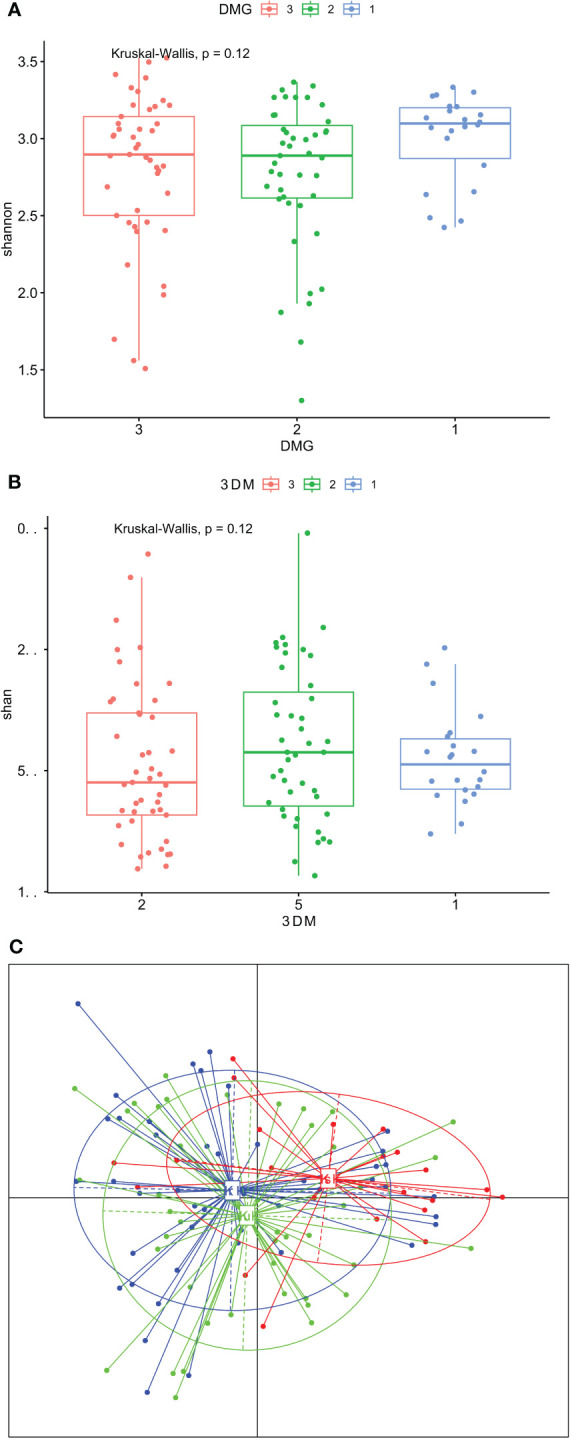
**(A–C)**. Gingival crevicular fluid microbiome composition of people with type 1 diabetes and healthy controls. **(A)** – Shannon diversity; **(B)** – Chao diversity; **(C)** - PCoA (beta diversity); group 1 – people with type 1 diabetes and gingivitis (G), group 2 – people with type 1 diabetes without gingivitis, group 3 – healthy controls.

GCF community composition did not differ between T1D cases and controls considering insignificant differences in the Shannon and Chao indices (**Figure 2A, B**), whereas there were borderline significant differences in the β-diversity (p =0.058, **Figure 2C**). In the first comparison between people with T1D without G and healthy controls, after adjusting for multiple comparisons, no differentiating taxa were identified. Next, we compared the GCF microbiomes of people with T1D and G to those with T1D and no gingival pathology. We found as many as 31 differential taxa at the adjusted p-value significance level when comparing people with T1D and G to those without G. All but one taxon, *Cutibacterium* spp. (p adjusted 0.04; FC -0.88), were overrepresented in participants with G (**Supplementary Table 2**). Third, people with T1D and G were compared with healthy controls. There were 38 differentially expressed taxa at the adjusted p-value significance level. All but one taxon, *Hemophilus* spp. (adjusted 0.026; FC 1-.79), were overrepresented in T1D participants with G (**Supplementary Table 3**).

A side-by-side comparison of the differential taxa between people with T1D and G and those with T1D without G vs. those with T1D and G and healthy controls is presented in **Table 1**. In total, 20 taxa were different in both comparisons: 11 were specific only for comparisons between people with T1D and G vs. those with T1D without G, and 17 were present only in comparisons between people with T1D and G and healthy controls. Most taxa were enriched in people with T1D with G, apart from *Cutibacterium* ssp., which was more abundant than that in people with T1D without G vs. those with T1D and G, and *Hemophilus* spp. which was enriched in healthy controls vs. in people with T1D and G (**Table 1**).

**Table 1:** Side-by-side comparison of differential taxa between people with T1D and gingivitis (G) and those with T1D without G vs. those with T1D and G vs. healthy controls.

Taxon	Differential species T1D with G vs. T1D without G	Differential species T1D with G vs. healthy controls
***Peptoanaerobacter* (100)**	X	
***Anaeroglobus* (100)**		X
***Bacteria*_unclassified (100)**	X	X
***Bacteroidales*_unclassified (100)**		X
***Bacteroides* (100)**		X
***Bacteroidia*_unclassified (100)**		X
***Campylobacter* (100)**	X	X
***Campylobacterales*_unclassified (100)**	X	X
***Clostridia*_unclassified (100)**	X	
***Clostridia*_vadinBB60_group_ge (100)**	X	X
***Cutibacterium* (100)**	X †	
***Defluviitaleaceae*_UCG-011 (100)**	X	
***Desulfobacterota*_unclassified (100)**	X	
***Desulfobulbus* (100)**	X	X
**Family_XIII_UCG-001 (100)**	X	
***Filifactor* (100)**	X	
***Firmicutes*_unclassified (100)**		X
***Fretibacterium* (100)**	X	
***Fusobacteriaceae*_unclassified (100)**	X	X
***Fusobacteriales*_unclassified (100)**	X	X
***Fusobacterium* (100)**	X	X
***Haemophilus* (100)**		X †
***Lachnospirales*_unclassified (100)**	X	X
***Lactobacillus* (100)**		X
***Muribaculaceae*_ge (100)**		X
***Mycoplasma* (100)**	X	
***Negativicutes*_unclassified (100)**		X
***Oscillospiraceae*_unclassified (100)**		X
***Peptoanaerobacter* (100)**		X
***Peptostreptococcaceae*_unclassified (100)**	X	X
***Peptostreptococcales*-*Tissierellales*_fa_unclassified (100)**	X	X
***Phocaeicola* (100)**	X	
***Prevotella* (100)**		X
***Prevotellaceae*_ge (100)**	X	
***Prevotellaceae*_NK3B31_group (100)**	X	X
***Prevotellaceae*_UCG-001 (100)**	X	X
***Prevotellaceae*_unclassified (100)**		X
***Proteobacteria*_unclassified (100)**		X
***Rikenellaceae*_RC9_gut_group (100)**	X	X
***Rikenellaceae*_unclassified (100)**	X	X
***Slackia* (100)**		X
***Spirochaetaceae*_unclassified (100)**	X	X
***Spirochaetota*_unclassified (100)**	X	X
***Synergistaceae*_unclassified (100)**	X	X
***Tannerella* (100)**	X	X
***Tannerellaceae*_unclassified (100)**		X
***Treponema* (100)**	X	X
***Veillonellaceae*_unclassified (100)**		X

The abundance of a taxon was higher in people with type 1 diabetes than in the compared group unless otherwise marked †.

T1D, type 1 diabetes; G, gingivitis.

X, A taxon was present in a subgroup.

Additionally, within the T1D group, those with HbA1c% in the first and fourth quartiles were compared, regardless of their gingival status. We further explored the association between the community structure of the GCF microbiome and HbA1c levels in people with T1D. There were no significant differences in the Shannon and Chao indexes and β-diversity (**Supplementary Figure 1A–C**). Two genera, Family_XIII_UCG-001 (padj 0.08; FC 2.53) and *Prevotellaceae*_YAB2003_group (p-adjusted 0.08; FC 2.25), tended to be overrepresented in the fourth quartile compared to the those in the first quartile of HbA1c after adjustment for multiple comparisons (**Supplementary Table 4**).

### 3.2 Metabolome

**Figure 3:**
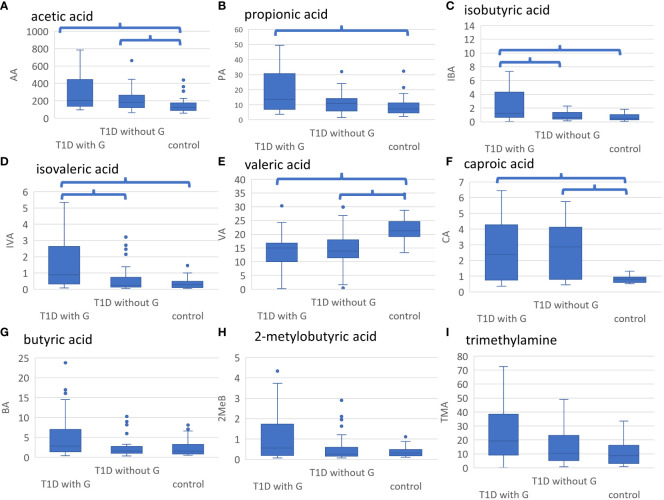
**(A-I)**. Results of metabolomic analyses. Healthy controls without gingivitis (G), people with T1D without G and those with T1D and G were compared. Significant differences after adjustment with p value <0.05 are marked. **(A)** AA was significantly higher in people with T1D and G and those with T1D without G vs. healthy controls, **(B)** PA was significantly higher in people with T1D and G vs. healthy controls, **(C)** IBA was significantly higher in people with T1D and G vs. those with T1D without G and healthy controls, **(D)** IVA was significantly higher in people with T1D and G vs. those with T1D without G and healthy controls, **(E)** VA was significantly higher in healthy controls vs. all people with T1D, **(F)** CA was significantly lower in healthy controls vs. all people with T1D, **(G)** BA tended to be higher in people with T1D and G vs. those with T1D without D and healthy controls (not statistically significant), **(H)** 2MeB tended to be higher in people T1D and G vs. those with T1D without G and healthy controls (not statistically significant), **(I)** TMA tended to be higher in people with T1D and G vs. those with T1D without G and healthy controls (not statistically significant). LA, lactic acid; AA, acetic acid; PA, propionic acid; IBA, isobutyric acid; BA, butyric acid; 2MeB, 2-metylobutyric acid; IVA, isovaleric acid; VA, valeric acid; ICA, isocaproic acid; CA, caproic acid; TMA, trimethylamine; GPA, glycerophosphorylcholine; A-H, concentration in mmol/l; I, concentration in umol/l.

When comparing three groups – healthy controls without G, people with T1D without G, and people with T1D and G, after adjusting for multiple comparisons, there were some significant differences in the concentrations of the selected metabolites (**Table 2**). Acetic acid concentration was higher in people with T1D than in healthy controls, regardless of the presence of G. Propionic acid was significantly higher in people with T1D and G than in healthy controls. Isobutyric and isovaleric acid levels were higher in people with T1D and G than those in the other two subgroups. In contrast, valeric acid was lower and caproic acid was higher in individuals with diabetes (regardless of gingival status) than those in healthy controls (**Figures 3A-E**). There were some borderline insignificant results for the selected SCFAs and TMA (**Figures 3F-H**). In the correlation analysis between HbA1c and SCFA concentrations in people with T1D, there was only one significant result for VA, but the correlation was weak (Spearman’s correlation coefficient -0.272, p=0.031; **Supplementary Table 5**).

**Table 2:** Results of metabolomic analyses.

Metabolite	T1D with G	T1D without G	Healthy controls	Adjusted p value
**Lactic acid [mmol/l]**	93.9 (62.6-180.4)	123.3 (39.8-177.6)	79.8 (55.8-148.3)	NS
**Acetic acid [mmol/l]**	206.6 (134.8-451.3)*	181.8 (122.0-264.1)#	125.9 (88.4-179.9)*#	<0.001
**Propionic acid [mmol/l]**	13.5 (7.0-32.4)*	10.9 (6.0-14.4)	7.2 (7.5-11.3)*	0.006
**Isobutyric acid [mmol/l]**	1.3 (0.6-4.4)*#	0.6 (0.4-1.5)*	0.5 (0.3-1.1)#	<0.001
**Butyric acid [mmol/l]**	2.9 (1.3-7.5)	1.6 (1.0-2.8)	1.6 (0.8-3.2)	0.09
**2-metylobutyric acid [mmol/l]**	0.6 (0.2-1.7)	0.3 (0.2-0.7)	0.3 (0.2-0.5)	0.068
**Isovaleric acid [mmol/l]**	0.9 (0.3-2.6)*#	0.3 (0.1-0.8)#	0.3 (0.1-0.5)*	0.03
**Valeric acid [mmol/l]**	15.0 (9.8-16.9)*	13.8 (11.2-18.3)#	21.3 (19.1-24.7)*#	<0.001
**Isocaproic acid [mmol/l]**	0.4 (0.1-1.0)	0.2 (0.1-0.6)	0.2 (0.1-0.4)	NS
**Caproic acid [mmol/l]**	2.4 (0.7-4.4)*	2.9 (0.8-4.2)#	0.8 (0.6-0.9)*#	<0.001
**Trimethylamine [umol/l]**	19.3 (7.8-38.6)	10.5 (5.1-38.6)	8.7 (19.3 (7.8-38.6)	0.059
**Betaine [umol/l]**	171.5 (56.3-482.2)	109.5 (73.0-304.3)	119.4 (37.2-209.0)	NS
**Glycerophosphorylcholine [umol/l]**	74.4 (41.0-108.5)	96.3 (55.9-156.8)	76.4 (47.5-142.7)	NS
**Choline [umol/l]**	351.1 (163.5-553.4)	323.8 (202.3-509.9)	432.3 (215.1-631.5)	NS
**Carnitine [umol/l]**	71.1 (34.7-111.8)	49.0 (29.6-73.9)2	47.7 (35.2-73.2)	NS

Data are presented median (interquartile range).

T1D, type 1 diabetes; G, gingivitis; NS, not significant.

*# significant difference in post-hoc analysis at p>0.05.

### 3.3 Integrated metagenomic–metabolomic analysis

**Figure 4:**
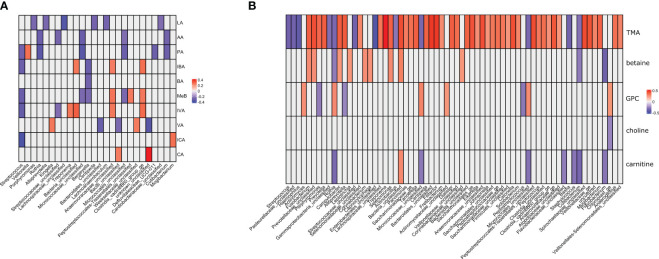
**(A, B)**. Metagenomic–metabolomic analyses between the identified bacterial taxa and selected metabolites concentrations. Correlation coefficients significant at p>0.05 are presented and color-coded. **(A)** lactic acid and short chain fatty acids. **(B)** trimethylamine and its metabolites. LA, lactic acid; AA, acetic acid; PA, propionic acid; IBA, isobutyric acid; BA, butyric acid; 2MeB, 2-metylobutyric acid; IVA, isovaleric acid; VA, valeric acid; ICA, isocaproic acid; CA, caproic acid; TMA, trimethylamine; GPA, glycerophosphorylcholine.

In the metagenomic–metabolomic analysis, we analyzed the correlations between the identified bacterial taxa and selected metabolite concentrations. The results are shown in **Figures 4A, B**.

There were 85 and 46 correlations with absolute value above 0.25 for amino acids and SCFAs, respectively. All correlations were statistically significant at a nominal p-value <0.05 (**Supplementary Table 6**). Among the amino acids, TMA had the strongest positive correlation with 20 correlations with coefficients above 0.4 (**Supplementary Table 6**). There were also two strong negative correlations (coefficient below -0.4). The correlations with SCFAs were of low strength, with only one absolute value above 0.4. Most correlations between SCFA concentrations and bacterial genera were weak or moderate. *Bergeyella* spp. were negatively correlated with BA, IBA, and MeB. *Kingella* spp. were positively correlated with VA. *Micrococcae* were negatively correlated with AA, PA, and MeB. *Rothia* spp. were negatively correlated with AA and PA. *Streptococcae* were negatively correlated with AA, PA, MeB, IBA, ICA, and IVA. *Veilonella* spp. were positively correlated with PA, and *Treponema* spp. with IVA. Trimethylamine was the only metabolite that showed moderate correlation with the selected bacterial genera. The strongest positive correlations were observed with *Treponema* spp., *Tanerella* spp., *Filifactor* spp., *Tanerella* spp., and *Porphyrimonas* spp. (**Table S6**). The greatest negative correlations were observed for *Streptococcus* spp., *Hemophilus* spp., *Rothia* spp., *Basfia* spp., and *Flavobacterium* spp.

## 4 Discussion

### 4.1 Microbiome

To date, most oral microbiome studies on people with T1D have included children or individuals with a wide range of metabolic controls. Furthermore, saliva is usually analyzed. To the best of our knowledge, this is the first study to investigate the GCF microbiome in people with T1D. In our study, we aimed to fill the knowledge gap in people with longstanding T1D treated with modern technologies, such as CSII, demonstrating good glycemic control.

In this study, we showed that intensively treated people with T1D with satisfactory glycemic control and non-diabetic individuals did not differ considering the Shannon and Chao indices, but β-diversity tended towards significant differences, especially pronounced for people with concomitant T1D and G. There were no differences within the T1D subgroup when comparing participants in the first and fourth quartile of HbA1c, that is, those with the best and worst metabolic control of diabetes. This is an important conclusion of the study. With relatively well-controlled diabetes, slight differences in glycemic control did not significantly affect the oral microbiome, which was comparable to that observed in people without diabetes. Despite good metabolic control of diabetes, people with T1D had a higher prevalence of mild G than healthy controls. People with T1D and G show apparent shifts in the GCF microbiome and metabolome, which have been associated with periodontitis.

In one study on the oral microbiome in children with metabolically stable T1D, differences between T1D participants and healthy children included reduced Shannon diversity in the T1D group. However, no differences in bacterial diversity have been reported in a cohort similar to that in the present study (51). When comparing participants after stratification by HbA1c level, the differences in microbiome composition were barely notable in our cohort, which is in line with other studies (17, 51). In contrast, another study investigated a cohort of people with severely uncontrolled T1D. Those with high HbA1c levels were characterized by significantly decreased Chao and Shannon indices and an increased Simpson index of the oral microbiome (52). A comparison between individuals with T1D without periodontal pathology and healthy controls did not reveal any significant differences in the GCF microbiome, with no apparent differentiating taxa between these two groups. This may suggest that in T1D population intensive treatment and good glycemic control along with the lack of oral pathology can increase the probability of preserving the “healthy” GCF microbiome in this population.

Previous studies comparing the oral microbiome in people with T1D and non-diabetic populations have reported some distinctions. They showed a significantly high abundance of *Streptococcus* spp., *Actinomyces* spp., and *Rothia* spp (17).. In another study, commensal *Streptococcus* spp., *Granulicatella* spp., *Rothia* spp., and *Rhodococcus* spp. were decreased in diabetic children as well as *Veillonella* spp. and *Prevotella* spp. However, these T1D children presented with severe glycemic dysregulation (52). Importantly, in studies comparing the oral microbiome between people with T1D with non-diabetics, a thorough assessment of oral health status has not been performed widely, in contrast to this study. However, the results of the selected reports are similar to ours (51). Within the T1D subgroup (regardless of gingival pathology), the abundance of *Anaerovoracaceae* and *Prevotellaceae* was higher in those with poor metabolic control. The higher prevalence of *Prevotella* in individuals with T1D and worse diabetes control may be associated with a higher risk of PD in this subgroup. A poor-quality, high-sugar diet in T1D has been reported to be associated with a high abundance of *Prevotella copri (* 53). This was linked to an altered periodontal status (54, 55). *Anaerovoracaceae* is another novel taxon that has been reported in oral microbiome studies. These genes were found to be enriched in individuals with diabetic retinopathy (56).

Additional analyses comparing individuals with T1D and G and those with only T1D or non-diabetic individuals with a healthy periodontium revealed some interesting differences. G seemed to be responsible for the majority of these discrepancies, as multiple taxa differentiated participants with G from those without such pathology, including some associated with various oral pathologies, such as *Fusobacterium* spp (57)., *Negativicutes* (58), *Prevotella* spp (54, 55)., *Tanerella* spp., and *Treponema* spp (59).. Previously, periodontally healthy diabetic participants had lower species richness than healthy controls, but also had higher loads of red complex species responsible for the development of periodontal pathology, potentially putting them at risk of developing periodontitis (59, 60). There is no consensus on whether oral microbiome diversity is higher (61) or lower (60) in people with diabetes and PD. Specifically, the subgingival tissue microbiome showed a relatively high abundance of *Leptotrichiaceae, Neisseriaceae, Lactobacillus, Corynebacterium*, *Pseudomonas, Saccharibacteria, Aggregatibacter, Neisseria, Gemella, Eikenella, Selenomonas, Actinomyces, Capnocytophaga, Fusobacterium, Veillonella, Streptococcus*, and *Actinomyces*. For *Filifactor, Prevotella*, and *Parvimonas*, low abundances were observed (59). To date, studies have mainly included individuals with periodontitis. However, in our study, the spectrum of PD was limited to mild cases of G. This and the good metabolic control of diabetes may have been one of the reasons for the discrepancies between our data and those of previous research.

A more detailed analysis of the differences in differentiating taxa between people with T1D and G and T1D without it vs. those with T1D and G and non-diabetic controls revealed some interesting observations. Comparisons within people with T1D omit the impact of diabetes on any potential differentiating taxa, showing, in our opinion, the core differences between those with and without G. Nevertheless, quantitatively, there were more differentiating taxa in the analysis of nondiabetics than in those with T1D. Qualitatively, some taxa that differentiated people with T1D and G vs. non-diabetics but their abundances were similar within people with T1D included *Bacteroides* spp., *Firmicutes* spp., and *Lactobacillus* spp. This suggests that individuals with T1D, even those without clinically visible signs of periodontal pathology, show specific shifts in the GCF microbiome, putting them at risk of developing PD.

### 4.2 Metabolome

SCFAs are formed from saturated aliphatic organic acids containing one–six carbon atoms (62). SCFAs are produced by gut microbes during fiber fermentation (63). Considering the gut microbiome, SCFAs have also been investigated as potential additives in the regular diet to positively influence insulin sensitivity, obesity, diabetes control, and immune modulation to counter autoimmune diseases (63, 64). SCFAs, as products of bacterial metabolism, can also be found in periodontal pockets; however, there are only singular studies on this subject (65). Bacteria known to produce SCFAs include *Porphyromonas gingivalis, Treponema denticola, Aggregatibacter actinomycetemcomitans, Prevotella intermedia*, and *Fusobacterium nucleatum* (66). GCF SCFAs produced locally in gingival pockets seem to play a role opposite to that in the gut and are considered responsible for local pathologies, such as PD. The complexity is added by data suggesting that specific nutrients that modify the level of SCFAs production in the gut can improve periodontal status (67, 68). Finally, not only are SCFAs produced by bacteria, but dietary SCFAs can also influence the microbiome composition, implicating complex bidirectional associations (33).

In our study, the concentrations of selected SCFAs, including acetic acid and caproic acid, were higher in people with T1D than in those without diabetes, whereas isobutyric and isovaleric acids were higher in people with T1D and G than in the remaining participants. However, the concentration of valeric acid was lower in people with diabetes than in healthy controls. These findings are in agreement with the results of the integrated metagenomic–metabolic analysis. For example, *Treponema* spp. were positively correlated with isovaleric acid concentrations, which were higher in participants with T1D and G. Interestingly, we did not identify differences in the abundance of *Streptococcus* spp. between T1D participants and healthy controls but identified negative correlations between *Streptococcus* spp. and acetic acid, isobutyric acid, isocaproic acid, and isovaleric acid concentrations. The levels of these metabolites were high in the T1D subgroup. This, in concurrence with the abovementioned findings in differential taxa between the studied groups, may suggest the first stage towards the development of an abnormal oral microbiome and, further, the emergence of PD.

Along with the early alterations in the microbiome discussed above, disturbances in metabolite concentrations may be treated as a marker of oral cavity acidification, an auxiliary predisposing factor for caries and PD (69). This is consistent with previous observations showing an abundance of acid-producing bacteria in individuals with diabetes. As this process progresses, it can lead to destabilization of the balance between *Streptococcus* spp. and the emergence of clinical oral pathology (70).

Acetate, derived primarily from microorganisms on the skin in the oral cavity and the gastrointestinal, urogenital, and respiratory tracts, has immunomodulatory effects (71). They are also involved in tissue development, nutrient absorption, and metabolism (72–74). Acetate plays a role in maintaining the intestinal barrier (75, 76). The metabolic processes affected by acetate include the accumulation of body fat, liver lipids, and cholesterol synthesis (77). Acetate in the gut is predominantly produced by *Prevotella* and *Bifidobacterium* spp (78).. Acetate is also used by *Firmicutes* to produce butyrate (79). The serum concentrations of acetate and propionate in individuals with T1D have been reported to be lower than those in individuals without diabetes (17). Importantly, the roles of serum and gut acetate seem to contradict that of oral acetate. Although serum acetate may protect against the emergence of anti-islet cell autoantibodies (80), local oral acetate administration may also promote periodontal pathology.

In type 2 diabetes, propionate acts locally on tissues, improving insulin sensitivity, suppressing cholesterol synthesis, and lowering the risk of cardiovascular disease (81). In a mouse model, gut integrity is ensured by the healthy commensalism of lactate- and butyrate-producing bacteria, with non-butyrate-producing bacteria preventing optimal mucin synthesis in individuals with type 1 diabetes (82). Butyrate has also been intensely investigated as a supplement to improve immune function, strength, and physical function and alleviate symptoms of gastrointestinal tract diseases (83).

We did not observe any changes in GCF butyrate concentrations between people with T1D and healthy controls. One possible explanation for this may be that the gingival pocket was the site of sampling in our study, as most studies have assessed its levels in the gut or serum. Moreover, none of the participants had PD. Nevertheless, we hypothesized that the observed alterations in SCFAs concentrations may reflect a predisposition towards the development of an early preclinical stage of PD.

A few studies that investigated oral propionate and butyrate have reported that their levels are increased in PDs, regardless of the diagnosis of diabetes (84). In participants with PD, the GCF concentrations of acetic, propionic, and butyric acids were positively correlated with *P. gingivalis*, *T. denticola, F. alocis*, *T. socraskii, F. nucleatum, and T. forsythia* (85, 86).

*In vitro* studies have shown that high concentrations of butyrate can induce apoptosis in gingival fibroblasts, leading to periodontal tissue damage (87, 88). High concentrations of lactic acid and a wide range of SCFAs, including acetic, propionic, butyric, and isovaleric acids, in GCF have been observed in patients with aggressive periodontitis. Interestingly, the probing depth and attachment loss were positively correlated with the concentrations of the selected SCFAs (85). As butyrate can have both detrimental and beneficial effects, this is likely attributed to the tissues where it is produced, i.e., where it acts locally, and its concentration (89).

The functional and physiological effects of isobutyric and 2-methylbutanoic acids are poorly understood. Valeric acid in the gut is produced by the microbial metabolism of lactic acid and propionic acids (90). A study of women with gestational diabetes (GDM) showed that the levels of isobutyric, isovaleric, valeric, and caproic acids were high in women with GDM (91). The authors linked high levels of inflammation to hyperglycemia and a dysbiotic gut microbiome in this population (91). Women with GDM have also been reported to have a high abundance of *Prevotella* spp., a potential caproic acid-producer, which leads to increased caproic acid production (92). Our findings on valeric, isovaleric, and caproic acids in the GCF of people with T1D are novel and partially in contrast with these reports, requiring further investigation and analysis in conjunction with other potential factors, such as diet or body composition.

Trimethylamine and its metabolites were not significantly different between the subgroups in our study, with TMA being the only metabolite nearing significance, with numerically higher concentrations in people with T1D and G. Trimethylamine levels are elevated in patients with PD (93). In our study, trimethylamine concentrations were positively correlated with typical red complex bacteria (*Tanerella* spp., *Treponema* spp., and *Fusobacterium* spp.) that are responsible for the development of PDs.

### 4.3 Limitations

The inclusion of only relatively young people with T1D with good glycemic control may be regarded as both a limitation and a strength. This is a homogeneous subgroup representative of a large portion of the T1D population. The percentage of well-controlled people with T1D within the entire T1D population will probably increase, at least in developed countries, with more common usage of advanced technologies, such as continuous glucose measurements and semi-automated or hybrid insulin pumps. However, our findings cannot be extrapolated to patients with poor metabolic control. Notably, our group was relatively young and free of advanced complications of diabetes. Thus, the results of our analysis cannot be extrapolated to older populations or individuals with advanced micro-and macrovascular complications of diabetes, such as renal failure and advanced cardiovascular diseases. We did not observe any cases of PD in our population, which may have affected our microbiome and metabolome findings. In this study, we focused on limited confounders related to microbiome and metabolome results. As more data regarding diet and body structure remain to be analyzed, the acquired picture may not be fully explained. Finally, measurements of fecal and serum SCFA levels may poorly reflect biologically active SCFA levels. Only up to 10% are found in these types of samples, as active SCFAs are constantly produced, utilized by microbial cross-feeding, or interact with host cells (17).

## 5 Conclusions

The GCF microbiome in intensively treated people with T1D with satisfactory glycemic control and healthy gingival tissues was similar to that in non-diabetic controls. People with T1D and G show clear shifts in the GCF microbiome and metabolome. In this cohort of people with T1D, HbA1c% did not have a significant impact on SCFA concentrations in the correlation analysis. By contrast, the GCF microbiome appeared to have a significant relationship with SCFAs. This suggests that despite good metabolic control of diabetes, people with T1D are susceptible to the development of PDs. This was demonstrated by early changes in the structure of the GCF microbiome and altered concentrations of selected metabolites in this environment.

To summarize, the identification of early changes in periodontal tissues by targeting microbiome and metabolome changes could potentially enable effective prevention and initial treatment of PD in people with T1D.

## Data availability statement

The data presented in the study are deposited in the NCBI BioProject repository, accession number ID: 1064953.

## Ethics statement

This study involving humans was approved by the Jagiellonian University Bioethics Committee (Komisja Bioetyczna Uniwersytetu Jagiellońskiego). The study was conducted in accordance with local legislation and institutional requirements. All the participants provided written informed consent to participate in this study.

## Author contributions

IG-M: Conceptualization, Funding acquisition, Investigation, Methodology, Project administration, Resources, Supervision, Writing – original draft, Writing – review & editing. MKa: Data curation, Formal analysis, Investigation, Validation, Visualization, Writing – original draft, Writing – review & editing. MD: Data curation, Formal analysis, Software, Validation, Visualization, Writing – review & editing. ES: Data curation, Formal analysis, Software, Validation, Visualization, Writing – review & editing. NŻ-L: Data curation, Formal analysis, Software, Validation, Visualization, Writing – review & editing. MKu: Data curation, Formal analysis, Software, Validation, Visualization, Writing – review & editing. TK: Methodology, Resources, Supervision, Writing – review & editing.
