# Supplementary material for: The interplay between gingival crevicular fluid microbiome and metabolomic profile in intensively treated people with type 1 diabetes - a combined metagenomic/metabolomic approach cross-sectional study
Source: Front Endocrinol (Lausanne). 2024 Feb 2;14:1332406. doi: 10.3389/fendo.2023.1332406 (PMC10871129; doi:10.3389/fendo.2023.1332406)
Supplement: Supplementary file 2 [file Table_1.docx]

**Supplementary file 1 – supplementary tables**

| Table S1. Basic characteristics of the study population. | | | | |
| --- | --- | --- | --- | --- |
|  | T1D with G (N=22) | T1D without G (N=43) | Healthy controls (N=45) | Adjusted P value |
| Age, years | 30 (23-34.5) | 25 (22-32) | 27 (24-30) | NS |
| Gender (female) | 9 (40.9%) | 17 (39.5%) | 18 (40.9% | NS |
| HbA1c, % | 7.4 (6.9-8.3) | 6.8 (6.5-7.7) | 5.1 (4.9-5.3) | <0.001* |
| T1D duration, years | 18 (9-22) | 14 (9-19) | - | NS |
| Missing teeth, N | 0 (0-0.3)* | 0# | 0*# | 0.017 |
| PI | 3.0 (2.0-3.0)* | 2.0 (1.0-3.0)# | 1.0 (1.0-2.8)*# | 0.002 |
| GI | 2.0 (1.0-2.0)* | 1.0 (0-1.0) | 0 (0-1.0)* | <0.001 |
| CAL min. 1mm, N | 20 (90.9%) | 17 (42.5%) | 20 (45.5%) | <0.001 |
| PPD, min. 3mm, N | 8 (38.1%) | 1 (2.4%) | 1 (2.4%) | <0.001 |
| TN, N |  |  |  | 0.038 |
| TN 1 | 0 | 5 (11.6%) | 7 (15.6%) |  |
| TN 2 | 20 (90.9%) | 38 (88.4%) | 38 (84.4%) |  |
| TN 3 | 2 (9.1%) | 0 | 0 |  |
| Data are presented as median (interquartile range) or number (%).  *significant difference at p<0.001 in post hoc analysis for T1D with G vs. healthy controls and T1D without G vs. healthy controls  T1D – type 1 diabetes, HbA1c – glycated hemoglobin, G – gingivitis, NS – not significant, PI – plaque index, GI -CAL – clinical attachement leve, PPD – pocket probing depth, TN – treatment needs; *#significant difference in post-hoc analysis at p<0.001 | | | | |

**The interplay between gingival crevicular fluid microbiome and metabolomic profile in intensively treated people with type 1 diabetes - a combined metagenomic/metabolomic approach**

| Table S2. Relative abundance of selected taxa at the genus level composition of people with T1D with gingivitis vs. those with T1D and no gingival pathology. | | | | | |
| --- | --- | --- | --- | --- | --- |
|  | baseMean | log2FoldChange | log2FoldChangeSE | Raw P value | Adjusted p value |
| *Peptoanaerobacter*(100) | 7,90591883 | 1,03150053 | 0,19935287 | 2,5345E-06 | 0,00072488 |
| *Rikenellaceae*_RC9_gut_group(100) | 47,0191741 | 1,68755579 | 0,38933417 | 5,363E-05 | 0,00323363 |
| *Spirochaetaceae*_unclassified(100) | 75,4346865 | 1,57572121 | 0,36007229 | 4,6344E-05 | 0,00323363 |
| *Desulfobacterota*_unclassified(100) | 6,5138683 | 0,39069293 | 0,0904511 | 5,6532E-05 | 0,00323363 |
| *Peptostreptococcales*-*Tissierellales*_fa_unclassified(100) | 6,60954167 | 0,77279963 | 0,17371826 | 3,5897E-05 | 0,00323363 |
| *Treponema*(100) | 2329,23287 | 1,55377498 | 0,37660205 | 0,00011035 | 0,00419071 |
| *Tannerella*(100) | 2035,59187 | 1,22505612 | 0,29231581 | 8,8272E-05 | 0,00419071 |
| *Fretibacterium*(100) | 118,393341 | 2,06676824 | 0,50737368 | 0,00013188 | 0,00419071 |
| *Desulfobulbus*(100) | 19,6165221 | 1,12594108 | 0,27514923 | 0,00012378 | 0,00419071 |
| *Lachnospirales*_unclassified(100) | 4,16260441 | 0,73872421 | 0,18406692 | 0,00016162 | 0,00462238 |
| *Bacteria*_unclassified(100) | 4856,74011 | 0,71278961 | 0,1805302 | 0,00020106 | 0,00470329 |
| *Clostridia*_vadinBB60_group_ge(100) | 10,4994922 | 1,36147759 | 0,34757118 | 0,00022311 | 0,00470329 |
| *Defluviitaleaceae*_UCG-011(100) | 9,541383 | 1,29848978 | 0,32814658 | 0,00019527 | 0,00470329 |
| Family_XIII_UCG-001(100) | 6,8210357 | 1,40257082 | 0,35892666 | 0,00023023 | 0,00470329 |
| *Peptostreptococcaceae*_unclassified(100) | 18,9805434 | 1,03618092 | 0,27329066 | 0,00033782 | 0,00637564 |
| *Spirochaetota*_unclassified(100) | 6,57557726 | 0,94094713 | 0,24926479 | 0,00035668 | 0,00637564 |
| *Mycoplasma*(100) | 29,6908062 | 1,52477928 | 0,41726199 | 0,0005272 | 0,00886942 |
| *Rikenellaceae*_unclassified(100) | 5,93961112 | 1,1575185 | 0,32236473 | 0,00064589 | 0,01026244 |
| *Filifactor*(100) | 387,191835 | 1,39637909 | 0,3961299 | 0,00079503 | 0,01136897 |
| *Synergistaceae*_unclassified(100) | 7,41063829 | 0,83893075 | 0,23763975 | 0,0007821 | 0,01136897 |
| *Campylobacterales*_unclassified(100) | 56,5705586 | 0,81073031 | 0,24021127 | 0,00126762 | 0,01726378 |
| *Fusobacterium*(100) | 77597,378 | 0,52830443 | 0,16560233 | 0,00221687 | 0,02881932 |
| *Clostridia*_unclassified(100) | 89,4937575 | 1,03977947 | 0,33272189 | 0,0026875 | 0,03321414 |
| *Prevotellaceae*_UCG-001(100) | 27,8015839 | 1,02069588 | 0,3279178 | 0,0027872 | 0,03321414 |
| *Prevotellaceae*_ge(100) | 7,60444648 | 0,48352549 | 0,15822564 | 0,00328847 | 0,03762011 |
| *Campylobacter*(100) | 26667,5294 | 0,50529054 | 0,1702762 | 0,00424019 | 0,04331053 |
| *Fusobacteriales*_unclassified(100) | 61,8214755 | 0,75314762 | 0,25355222 | 0,0042052 | 0,04331053 |
| *Prevotellaceae*_NK3B31_group(100) | 5,39415884 | 0,81651989 | 0,27513382 | 0,00423726 | 0,04331053 |
| *Phocaeicola*(100) | 6,15108702 | 0,33894482 | 0,11494123 | 0,00447074 | 0,0440907 |
| *Cutibacterium*(100) | 98,4670868 | -0,8896153 | 0,30607919 | 0,00503919 | 0,04666577 |
| *Fusobacteriaceae*_unclassified(100) | 115,329191 | 0,73041986 | 0,25142242 | 0,00505818 | 0,04666577 |
| T1D – type 1 diabetes, baseMean - the average of the normalized count values, divided by size factors, taken over all samples; log2FC - log2 fold change between the groups; lfcSE - standard error of the log2FC estimate; padj - Benjamini–Hochberg-adjusted p-value | | | | | |

| Table S3. Relative abundance of selected taxa at the genus level composition of T1D participants with gingivitis vs. healthy controls. | | | | | |
| --- | --- | --- | --- | --- | --- |
|  | baseMean | log2FoldChange | lfcSE | Raw P value | Adjusted p value |
| *Bacteria*_unclassified(100) | 2570,5879 | 1,86793613 | 0,37438337 | 2,3584E-06 | 0,00033253 |
| *Fusobacteriaceae*_unclassified(100) | 36,7555245 | 2,55906929 | 0,53073224 | 4,7336E-06 | 0,00044496 |
| *Campylobacterales*_unclassified(100) | 20,5949742 | 2,4768395 | 0,57395299 | 3,5656E-05 | 0,00251377 |
| *Fusobacteriales*_unclassified(100) | 20,0296276 | 2,65349885 | 0,64883614 | 8,3898E-05 | 0,00473183 |
| *Rikenellaceae*_RC9_gut_group(100) | 18,344748 | 3,47645615 | 0,87056712 | 0,00011978 | 0,00562977 |
| *Tannerella*(100) | 1198,68179 | 2,41291855 | 0,61690362 | 0,0001615 | 0,00579657 |
| *Anaeroglobus*(100) | 8,26358641 | 2,86257254 | 0,73280077 | 0,00016444 | 0,00579657 |
| *Fusobacterium*(100) | 49248,2132 | 1,32394794 | 0,3617561 | 0,00039379 | 0,00790734 |
| *Treponema*(100) | 1434,48956 | 2,80281593 | 0,75437632 | 0,00032444 | 0,00790734 |
| *Firmicutes*_unclassified(100) | 490,198228 | 1,32675951 | 0,35411468 | 0,0002907 | 0,00790734 |
| *Rikenellaceae*_unclassified(100) | 0,89419982 | 3,14581686 | 0,86375328 | 0,00041873 | 0,00790734 |
| *Tannerellaceae*_unclassified(100) | 2,68576313 | 1,58630051 | 0,43179137 | 0,00037514 | 0,00790734 |
| *Prevotellaceae*_NK3B31_group(100) | 1,17218361 | 2,45919482 | 0,67020614 | 0,00038099 | 0,00790734 |
| *Spirochaetota*_unclassified(100) | 0,95839127 | 2,88832514 | 0,79743533 | 0,00044864 | 0,00790734 |
| *Lachnospirales*_unclassified(100) | 3,27912203 | 1,10177964 | 0,30361189 | 0,00043812 | 0,00790734 |
| *Bacteroides*(100) | 3,46941702 | 0,94595974 | 0,26385111 | 0,00050902 | 0,0084207 |
| *Prevotellaceae*_UCG-001(100) | 11,1842537 | 2,51298086 | 0,70721186 | 0,00056735 | 0,0084207 |
| *Negativicutes*_unclassified(100) | 0,55439897 | 2,39256418 | 0,6725375 | 0,0005594 | 0,0084207 |
| *Bacteroidales*_unclassified(100) | 503,500504 | 1,66206899 | 0,47417918 | 0,00066783 | 0,00941636 |
| *Peptoanaerobacter*(100) | 4,21577205 | 1,77921515 | 0,52310753 | 0,00094415 | 0,01267854 |
| *Prevotella*(100) | 26620,593 | 1,37314843 | 0,40590405 | 0,00100279 | 0,01285391 |
| *Synergistaceae*_unclassified(100) | 3,68696714 | 1,76030603 | 0,52437567 | 0,00109192 | 0,01338793 |
| *Clostridia*_vadinBB60_group_ge(100) | 7,70758834 | 2,2101151 | 0,6640735 | 0,00119953 | 0,01409449 |
| *Peptostreptococcales*-Tissierellales_fa_unclassified(100) | 4,23108154 | 1,37848068 | 0,42016784 | 0,00139793 | 0,01576867 |
| *Prevotellaceae*_unclassified(100) | 32594,3649 | 1,31494198 | 0,40337575 | 0,00149516 | 0,01607523 |
| *Oscillospiraceae*_unclassified(100) | 3,3245378 | 0,67855485 | 0,20873526 | 0,00153912 | 0,01607523 |
| *Spirochaetaceae*_unclassified(100) | 48,5286637 | 2,65694827 | 0,82592781 | 0,00171449 | 0,01667191 |
| *Lactobacillus*(100) | 4,95814216 | 1,92923438 | 0,59792163 | 0,00166275 | 0,01667191 |
| *Slackia*(100) | 8,0405657 | 1,56526483 | 0,4944493 | 0,00201563 | 0,01894692 |
| *Campylobacter*(100) | 17138,0387 | 1,23815256 | 0,39243683 | 0,002084 | 0,01895767 |
| *Veillonellaceae*_unclassified(100) | 615,627948 | 1,73700716 | 0,55549875 | 0,00227516 | 0,02004988 |
| *Muribaculaceae*_ge(100) | 1,60574353 | 1,23943094 | 0,40312666 | 0,00267566 | 0,02224792 |
| *Peptostreptococcaceae*_unclassified(100) | 14,4910151 | 1,79093609 | 0,58265885 | 0,00268237 | 0,02224792 |
| *Haemophilus*(100) | 74230,5603 | -1,79861651 | 0,59760676 | 0,00326173 | 0,02628022 |
| *Bacteroidia*_unclassified(100) | 301,335919 | 1,97865511 | 0,68542124 | 0,00470966 | 0,03589526 |
| *Desulfobulbus*(100) | 4,3599725 | 2,32733826 | 0,80515828 | 0,00465774 | 0,03589526 |
| *Proteobacteria*_unclassified(100) | 15,4927695 | 1,4431233 | 0,51499822 | 0,00602638 | 0,04472209 |
| T1D – type 1 diabetes, baseMean - the average of the normalized count values, divided by size factors, taken over all samples; log2FC - log2 fold change between the groups; lfcSE - standard error of the log2FC estimate; padj - Benjamini–Hochberg-adjusted p-value | | | | | |

| Table S4. Relative abundance of selected taxa at the genus level composition of T1D participants with HbA1c in the first and fourth quartile. | | | | | |
| --- | --- | --- | --- | --- | --- |
|  | log2FoldChange | baseMean | lfcSE | Raw P value | Adjusted p value |
| Family_XIII_UCG-001(100) | 2,5328823 | 30,1619977 | 0,62956723 | 0,00032811 | 0,08780485 |
| *Prevotellaceae*_YAB2003_group(100) | 2,25190091 | 6,57933215 | 0,59122842 | 0,00059731 | 0,08780485 |
| *Synergistaceae*_unclassified(100) | 2,29978149 | 8,8632793 | 0,6563489 | 0,0013781 | 0,1350534 |
| T1D – type 1 diabetes, baseMean - the average of the normalized count values, divided by size factors, taken over all samples; log2FC - log2 fold change between the groups; lfcSE - standard error of the log2FC estimate; padj - Benjamini–Hochberg-adjusted p-value | | | | | |

| Table S5. Correlations between HbA1c%, lactic acid and SCFAs in people with T1D. | | | |
| --- | --- | --- | --- |
| Metabolite | Correlation* | | p |
| Lactic acid | | 0.088 | NS |
| Acetic acid | | -0.16 | NS |
| Propionic acid | | 0.108 | NS |
| Isobutyric acid | | 0.227 | NS |
| Butyric acid | | 0.175 | NS |
| 2-metylobutyric acid | | 0.218 | NS |
| Isovaleric acid | | 0.145 | NS |
| Valeric acid | | -0.272 | 0.031 |
| Isocaproic acid | | 0.284 | NS |
| Caproic acid | | 0.044 | NS |
| Trimethylamine | | 0.084 | NS |
| Betaine | | -0.108 | NS |
| Glycerophosphorylcholine | | -0.052 | NS |
| Choline | | 0.047 | NS |
| Carnitine | | -0.080 | NS |
| *Spearman’s correlations coefficients are presented.  SCFAs – short chain fatty acids, HbA1c% – glycated hemoglobin, T1D – type 1 diabetes | | | |

| Table S6. Metagenomic-metabolomic analyses between the identified bacterial taxa and selected metabolites concentrations. Only significant correlations are presented. | | |
| --- | --- | --- |
| Metabolite | Bacterial taxa | Correlation coefficient |
| LA | *Alloprevotella*(100) | -0,26046805 |
| IBA | *Anaerovoracaceae*_unclassified(100) | 0,25020218 |
| IVA | *Anaerovoracaceae*_unclassified(100) | 0,26633673 |
| MeB | *Anaerovoracaceae*_unclassified(100) | 0,27277103 |
| CA | *Bacilli*_unclassified(100) | 0,2557597 |
| VA | *Bacilli*_unclassified(100) | -0,26414657 |
| IBA | *Bacteria*_unclassified(100) | 0,26894677 |
| IVA | *Bacteria*_unclassified(100) | 0,31850644 |
| VA | *Bacteroidales*_unclassified(100) | -0,30937428 |
| BA | *Bergeyella*(100) | -0,28265026 |
| IBA | *Bergeyella*(100) | -0,30357156 |
| MeB | *Bergeyella*(100) | -0,25932161 |
| LA | *Cardiobacteriaceae*_unclassified(100) | -0,25126141 |
| LA | *Centipeda*(100) | -0,27625765 |
| IBA | *Clostridia*_vadinBB60_group_ge(100) | 0,27833951 |
| IVA | *Clostridia*_vadinBB60_group_ge(100) | 0,29251667 |
| MeB | *Clostridia*_vadinBB60_group_ge(100) | 0,26565986 |
| AA | *Cutibacterium*(100) | -0,28458999 |
| PA | *Cutibacterium*(100) | -0,29453548 |
| PA | *Defluviitaleaceae*_UCG-011(100) | -0,29626087 |
| VA | *Kingella*(100) | 0,26727528 |
| LA | *Lachnoanaerobaculum*(100) | -0,27612944 |
| LA | *Lachnospiraceae*_unclassified(100) | -0,36112049 |
| AA | *Micrococcaceae*_unclassified(100) | -0,30833371 |
| MeB | *Micrococcaceae*_unclassified(100) | -0,25442589 |
| PA | *Micrococcaceae*_unclassified(100) | -0,35196554 |
| AA | *Micrococcales*_unclassified(100) | -0,25038418 |
| MeB | *Micrococcales*_unclassified(100) | -0,26209135 |
| PA | *Micrococcales*_unclassified(100) | -0,30313214 |
| ICA | *Mogibacterium*(100) | 0,28230822 |
| MeB | *Peptostreptococcales*-*Tissierellales*_unclassified(100) | 0,25646255 |
| LA | *Porphyromonas*(100) | -0,2618082 |
| AA | *Rothia*(100) | -0,30103534 |
| PA | *Rothia*(100) | -0,34427373 |
| AA | *Streptococcaceae*_unclassified(100) | -0,27327506 |
| IVA | *Streptococcaceae*_unclassified(100) | -0,25246066 |
| IBA | *Streptococcus*(100) | -0,29631104 |
| ICA | *Streptococcus*(100) | -0,3739306 |
| IVA | *Streptococcus*(100) | -0,31753377 |
| MeB | *Streptococcus*(100) | -0,30077557 |
| PA | *Streptococcus*(100) | -0,28298431 |
| IVA | *Treponema*(100) | 0,26849711 |
| CA | unknown_unclassified(100) | 0,41404333 |
| VA | unknown_unclassified(100) | -0,35219758 |
| PA | *Veillonella*(100) | 0,27901423 |
| VA | *Weeksellaceae*_unclassified(100) | 0,26060626 |
|  |  |  |
| TMA | *Actinobacillus*(100) | -0,28972658 |
| GPC | *Actinomyces*(100) | 0,27194498 |
| GPC | *Actinomycetaceae*_unclassified(100) | 0,25822102 |
| GPC | *Alloprevotella*(100) | -0,25603446 |
| TMA | *Alloprevotella*(100) | 0,41157371 |
| TMA | *Anaeroglobus*(100) | 0,35845358 |
| TMA | *Anaerovoracaceae*_ge(100) | 0,41934798 |
| TMA | *Anaerovoracaceae*_unclassified(100) | 0,43772915 |
| TMA | *Atopobiaceae*_unclassified(100) | 0,25089992 |
| karnityna | *Bacteria*_unclassified(100) | 0,29113912 |
| betaina | *Bacteria*_unclassified(100) | 0,31261254 |
| TMA | *Bacteria*_unclassified(100) | 0,44065358 |
| TMA | *Bacteroidales*_unclassified(100) | 0,47868514 |
| TMA | *Bacteroidia*_unclassified(100) | 0,39415939 |
| TMA | *Basfia*(100) | -0,35691314 |
| betaina | *Campylobacter*(100) | 0,25382263 |
| TMA | *Campylobacter*(100) | 0,4049342 |
| betaina | *Capnocytophaga*(100) | 0,26523574 |
| TMA | *Catonella*(100) | 0,43371627 |
| TMA | *Centipeda*(100) | 0,39528944 |
| cholina | *Chloroplast*_ge(100) | -0,25524853 |
| GPC | *Chloroplast*_ge(100) | 0,258362 |
| TMA | *Clostridia*_unclassified(100) | 0,36092542 |
| TMA | *Clostridia*_vadinBB60_group_ge(100) | 0,26001752 |
| betaina | *Corynebacteriaceae*_unclassified(100) | 0,25732142 |
| betaina | *Corynebacterium*(100) | 0,27109473 |
| karnityna | *Cutibacterium*(100) | -0,33808264 |
| TMA | *Cutibacterium*(100) | -0,30336866 |
| betaina | *Cutibacterium*(100) | -0,27204059 |
| TMA | *Dialister*(100) | 0,40637977 |
| TMA | *Eikenella*(100) | 0,2720342 |
| TMA | *Enterobacterales*_unclassified(100) | -0,46169616 |
| TMA | *Filifactor*(100) | 0,47772054 |
| TMA | *Firmicutes*_unclassified(100) | 0,25183533 |
| karnityna | *Flavobacteriaceae*_unclassified(100) | -0,27214267 |
| betaina | *Flavobacterium*(100) | -0,36938675 |
| karnityna | *Flavobacterium*(100) | -0,2916176 |
| TMA | *Fretibacterium*(100) | 0,2853135 |
| betaina | *Fusobacterium*(100) | 0,2783351 |
| TMA | *Fusobacterium*(100) | 0,40138148 |
| TMA | *Gammaproteobacteria*_unclassified(100) | -0,30916223 |
| TMA | *Haemophilus*(100) | -0,41482357 |
| TMA | *Johnsonella*(100) | 0,25197443 |
| TMA | Lachnospiraceae_unclassified(100) | 0,34073483 |
| betaina | *Leptotrichia*(100) | 0,25500395 |
| TMA | *Micrococcaceae*_unclassified(100) | -0,33154822 |
| karnityna | *Micrococcaceae*_unclassified(100) | -0,28263438 |
| GPC | *Micrococcaceae*_unclassified(100) | 0,30418222 |
| TMA | *Micrococcales*_unclassified(100) | -0,32248332 |
| karnityna | *Micrococcales*_unclassified(100) | -0,29749814 |
| GPC | *Micrococcales*_unclassified(100) | 0,28054415 |
| TMA | *Mogibacterium*(100) | 0,38569231 |
| TMA | *Parvimonas*(100) | 0,3215618 |
| TMA | *Pasteurellaceae*_unclassified(100) | -0,35742678 |
| TMA | *Peptococcus*(100) | 0,33084402 |
| TMA | *Peptostreptococcaceae*_ge(100) | 0,35299455 |
| TMA | *Peptostreptococcales*-*Tissierellales*_unclassified(100) | 0,44218818 |
| GPC | *Peptostreptococcus*(100) | -0,28599899 |
| GPC | *Porphyromonas*(100) | -0,26535506 |
| TMA | *Porphyromonas*(100) | 0,37179491 |
| TMA | *Prevotella*(100) | 0,40296565 |
| betaina | *Prevotellaceae*_unclassified(100) | 0,27010394 |
| TMA | *Prevotellaceae*_unclassified(100) | 0,43844041 |
| TMA | *Rothia*(100) | -0,32592989 |
| karnityna | *Rothia*(100) | -0,32513076 |
| betaina | *Rothia*(100) | -0,27686797 |
| GPC | *Rothia*(100) | 0,28622314 |
| TMA | *Saccharimonadaceae*_ge(100) | 0,35491658 |
| TMA | *Saccharimonadaceae*_unclassified(100) | 0,34033543 |
| TMA | *Saccharimonadales*_ge(100) | 0,42328379 |
| TMA | *Saccharimonadales*_unclassified(100) | 0,33317898 |
| TMA | *Selenomonadaceae*_unclassified(100) | 0,29673354 |
| betaina | *Selenomonas*(100) | 0,28067869 |
| TMA | *Selenomonas*(100) | 0,39043212 |
| TMA | *Solobacterium*(100) | 0,29893794 |
| TMA | *Spirochaetaceae*_unclassified(100) | 0,44193677 |
| karnityna | *Staphylococcus*(100) | -0,29347427 |
| TMA | *Streptococcaceae*_unclassified(100) | -0,35590086 |
| TMA | *Streptococcus*(100) | -0,35204529 |
| TMA | *Tannerella*(100) | 0,40022248 |
| TMA | *Treponema*(100) | 0,52054316 |
| TMA | uncultured(100) | 0,3029777 |
| TMA | *Veillonellaceae*_ge(100) | 0,42831223 |
| TMA | *Veillonellaceae*_unclassified(100) | 0,36308201 |
| TMA | *Veillonellales*-*Selenomonadales*_unclassified(100) | 0,30751619 |
| baseMean - the average of the normalized count values, divided by size factors, taken over all samples; log2FC - log2 fold change between the groups; lfcSE - standard error of the log2FC estimate; padj - Benjamini–Hochberg-adjusted p-value | | |


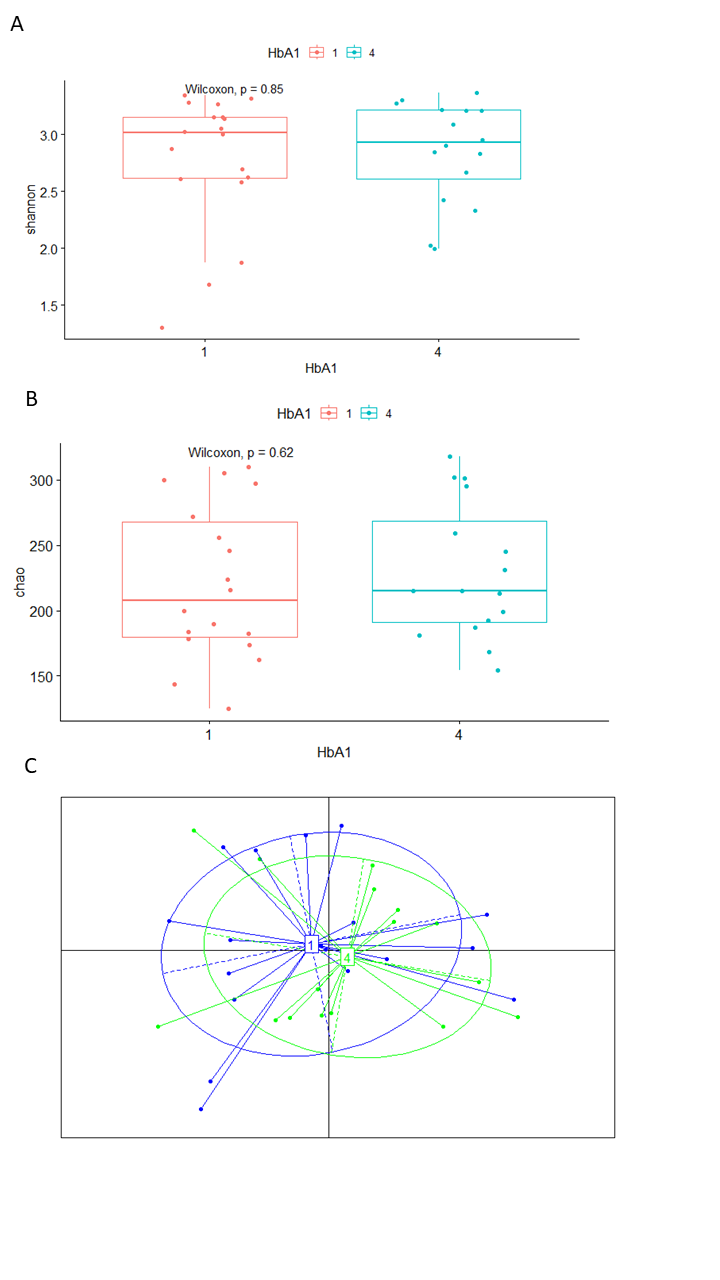


Figure S1. *Gingival crevicular fluid microbiome composition of type 1 diabetes participants with HbA1c in the 1^st^ and 4^th^ quartile. A – Shannon diversity; B – Chao diversity; C - PCoA (beta diversity); HbA1c% – glycated hemoglobin; 1 – first quartile of HbA1c%; 4 – fourth quartile of HbA1c%*
